# Supplementary material for: MetaRibo-Seq measures translation in microbiomes
Source: Nat Commun. 2020 Jun 29;11:3268. doi: 10.1038/s41467-020-17081-z (PMC7324362; doi:10.1038/s41467-020-17081-z)
Supplement: Supplementary file 10 — Supplementary Data 7 [file 41467_2020_17081_MOESM10_ESM.zip › File2/Confidence_High_Taxonomy/367898_out.krona.html]

Javascript must be enabled to view this page.

members
magnitude
magnitudeUnassigned
count
unassigned
taxon
rank

367898\_out

36

superkingdom
36
2

976
36
phylum

200643
36
class

36
171549
order

815
36
family

1
36
816

SRS021948\_contig\_number\_contig-100\_3499.204748
genus


SRS011405\_contig\_number\_8819SRS013940\_contig\_number\_14636SRS014948\_contig\_number\_4163SRS015960\_contig\_number\_8133SRS016629\_contig\_number\_contig-100\_465.28334SRS018836\_contig\_number\_15074SRS019286\_contig\_number\_contig-100\_198.199SRS019445\_contig\_number\_28122SRS019808\_contig\_number\_154SRS020869\_contig\_number\_28263SRS045004\_contig\_number\_1314SRS045645\_contig\_number\_3186SRS046369\_contig\_number\_6263SRS049959\_contig\_number\_6019SRS049995\_contig\_number\_15564SRS050422\_contig\_number\_36724SRS056259\_contig\_number\_21302SRS056273\_contig\_number\_12513SRS075078\_contig\_number\_19549SRS076756\_contig\_number\_contig-100\_366.134390SRS076976\_contig\_number\_5453SRS101376\_contig\_number\_3904SRS1041137\_contig\_number\_20080SRS1055043\_contig\_number\_21042SRS144506\_contig\_number\_44285SRS144537\_contig\_number\_contig-100\_243.250565SRS147139\_contig\_number\_553SRS148196\_contig\_number\_39864SRS148319\_contig\_number\_contig-100\_2296.89323SRS148511\_contig\_number\_18227SRS893369\_contig\_number\_2607
species
329854
31

246787
1

SRS1041145\_contig\_number\_3722
species

species

SRS1055099\_contig\_number\_13667SRS142599\_contig\_number\_3716
2
817

species

SRS104636\_contig\_number\_27180
818
1
